# Analysis of Non-Genetic Factors Affecting Wood’s Model of Daily Milk Fat Percentage of Holstein Cattle

**DOI:** 10.3390/vetsci9040188

**Published:** 2022-04-14

**Authors:** Fuzhen Zhou, Yan Liang, Abdelaziz Adam Idriss Arbab, Mingxun Li, Zhangping Yang, Niel A. Karrow, Yongjiang Mao

**Affiliations:** 1 College of Animal Science and Technology, Yangzhou University, Yangzhou 225009, China; zfzstrive@163.com (F.Z.); mz120181016@yzu.edu.cn (Y.L.); dh16012@stu.yzu.edu.cn (A.A.I.A.); limingxun@yzu.edu.cn (M.L.); yzp@yzu.edu.cn (Z.Y.); 2 Biomedical Research Institute, Darfur College, Nyala 63313, Sudan; 3 Center for Genetic Improvement of Livestock, Department of Animal Biosciences, University of Guelph, Guelph, ON N1G 2W1, Canada; nkarrow@uoguelph.ca

**Keywords:** wood lactation curve model, milk fat percentage, non-genetic factors, holstein cattle

## Abstract

This research paper aimed to explore the characteristics of Holstein cattle’s milk fat percentage lactation curve and its influencing factors. The Wood model was used for fitting the lactation curve of 398,449 DHI test-day milk fat percentage records of Holstein cows from 2018 to 2020 in 12 dairy farms in Jiangsu province, and the influencing factors—including farm size, parity, calving season, calving interval, and 305-days milk production—on the parameters of the lactation curve were analyzed. The results showed that the non-genetic factors such as dairy farm size, calving season, parity, calving interval, and 305-days milk yield have a significant impact on milk fat percentage (*p* < 0.01); the average *R*^2^ of the daily milk fat percentage curve was 0.9699; the lowest milk fat percentage was 3.54%; the time to reach the lowest milk fat percentage was 126 days; and the persistence of milk fat percentage was 3.59%. All of these factors explored in this study fit at different levels above 0.96. The Wood model performed well in the fitting and analysis of the milk fat percentage curve of Holstein cattle in Jiangsu Province. This study provides a reference for improving the milk fat percentage of Holstein cattle.

## 1. Introduction

Milk fat is a kind of high-quality natural fat, which is synthesized and secreted by dairy cow mammary epithelial cells. It is an important nutritional component of milk and dairy products. The content of fat in milk will significantly affect the price of milk, and milk with high milk fat content is preferred. Lactation curve is a mathematical model describing the variation of milk yield during lactation, which is applied to predict lactation traits, potential genetic estimation, breeding stock selection, etc. Related studies have shown that milk fat percentage decreases gradually in the early stage of lactation, reaches the lowest point after a certain period of time, and gradually increases over time, showing rhythmic changes in different stages of lactation [1,2,3]. Many scholars have analyzed, described and quantified this law of change by establishing or drawing lessons from various mathematical models. Previous studies have shown that the Wood incomplete gamma function model (Wood model), the Nelder model, the Wilmink model, the Ali–Schaeffer model, and the Dijkstra model have good fitting effects on the lactation curve [4,5,6,7,8,9,10,11]. Compared with the Wood model, most of the models are more complex, the number of estimated parameters is increased, and the amount of data required is larger, therefore they may be difficult to calculate [12]. Sun et al. [13] found that the Wood model was suitable for fitting the milk fat percentage curve of Holstein cattle in the Yangtze River Delta. Mao et al. [14] found that the fitting degree of the Wood model to the lactation curve of southern Chinese Holstein cattle was more than 0.99.

Related studies have shown that the parameters of the dairy cow lactation curve model are affected by many non-genetic factors, such as dairy farm size, parity, calving season, etc, [15]. The changes in these factors must have a certain influence on the lactation curve of dairy cows’ daily milk fat percentage. Previous studies have only focused on the calculation of variance components and genetic parameters of dairy farm size, parity and calving season on milk, and there have been few reports on the effect of the dairy cows’ lactation [16,17,18]. Therefore, this study set out to use the Wood model to fit the curve of the daily milk fat percentage of Holstein cattle in 12 farms in Jiangsu Province from 2018 to 2020. The changing trend of all data was described quantitatively, and the effects of non-genetic factors such as dairy farm size, parity, calving season, calving interval, and 305-days milk yield on the parameters of the daily milk fat percentage Wood lactation curve of Holstein cattle were analyzed. The aim of this study was to provide referential and technical support for the breeding process and scientific feeding and management of Holstein cattle in Jiangsu Province.

## 2. Materials and Methods

### 2.1. Farm and Animal Information

The 12 dairy herds in the study were located in Jiangsu Province. The farms belonged to the monsoon climate area, with a mild climate and four distinct seasons. The annual average temperature was between 13.6 °C and 16.1 °C, and the annual precipitation was 704–1250 mm. The calving season was classified by average climate variables, as spring (March to May), summer (June to August), autumn (September to November) and winter (December to February next year).

All the dairy farm cattle were raised in the free stall, with three feeding and milking times per day, and all were fed with Total Mixed Ration (TMR). Dairy cows were divided into different groups in dairy farms. The formula for one of the farms is shown in Table 1.

### 2.2. Data Source

A total of 580,025 Dairy Herd Improvement (DHI) records of Holstein cattle, including cow number, determination date, calving season, parity, calving interval, lactation days, milk fat percentage, and 305-days milk yield were collected from 12 dairy farms in Jiangsu Province from 2018 to 2020. Extremely abnormal data was screened and eliminated to ensure the accuracy of the final results. The screening criteria were as follows: the number of births was 1–5 parities, the number of days of lactation was less than 305 days, and the milk fat percentage was 1–7%. After screening, the total number of qualified records was 398,449.

### 2.3. Statistical Analysis

The change in daily milk fat percentage was measured by the Wood incomplete gamma function model. The basic model is as follows:

$$Y_{t}=at^{-b}e^{ct}$$

where *t* represents the month of lactation, *Y_t_* represents the milk fat percentage of time *t*, and *a*, *b* and *c* are the model parameters. Parameter *a* represents the lactation potential, *c* represents the speed at which the milk fat percentage reaches the lowest point, and *b* represents the percentage at which the milk fat percentage rises from the lowest point [19]. The expressions of *a*, *b* and *c* are all “mean ± standard error”. In curve fitting, the initial values of each parameter are determined by the results calculated by Olori et al. [20]. The parameters calculated in the first step (*a*, *b*, and *c*), along with the following three formulas, are used to calculate the secondary parameters:

*T_min_* = *b*/*c*

*Y_min_* = *a*(*b*/*c*)^−*b*^
*e^b^*

*Per* = −(*b* + 1) *lnc*

In the above formula, *T_min_* is the number of days to reach the lowest milk fat percentage; *Y_min_* is the lowest value of milk fat percentage; and *Per* is the lactation persistence of milk fat. Firstly, all the milk fat percentage data were sorted out in Microsoft Excel (2021), and then fitted with the SPSS (Ver 26.0) nonlinear regression subroutine. The fitting effect was evaluated by the degree of fit (*R*^2^) and the mean square of error. The least-square model was used to analyze the effects of non-genetic factors such as dairy farm size, parity, calving season, calving interval, and 305-days milk yield on the milk fat percentage of dairy cows. The model can be represented as follows:

*Y_ijklmn_* = *μ* + *F_i_* + *P_j_* + *S_k_* + *D_l_* + *N_m_* + *e_ijklmn_*

where *Y_ijklmn_* is the observed value of milk fat percentage; *F_i_* is the fixed effect of dairy farm size; *P_j_* is the fixed effect of parity; *S_k_* is the fixed effect of calving season; *D_l_* is the fixed effect of calving interval; *N_m_* is the fixed effect of 305-days milk yield; and *e_ijklmn_* is a residual random effect. The multiple comparisons between different levels of factors were made by Duncan’s method. The lactation month was divided into 1 lactation month every 30 days, and more than 300 days was the last lactation month, a total of 11 lactation months. The significance level was defined as follows: *p* ≤ 0.01, that the difference was extremely significant; *p* ≤ 0.05, that the difference reached a significant level; *p* > 0.05, that the difference was not significant. According to the above factors and levels affecting the daily milk fat percentage, the corresponding data were selected, the Wood lactation curve was fitted by the method mentioned above, and the corresponding parameters were calculated.

## 3. Results

### 3.1. Effects of Different Factors on Milk Fat Percentage of Holstein Cattle

Table 2 shows the basic situation of each parity of dairy cows’ milk fat percentage. The dairy farm size, parity, calving season, calving interval, and 305-days milk yield significantly impacted the milk fat percentage of Holstein cattle (Table 3, *p* < 0.01). The Holstein cow in the dairy farm with 2001 to 5000 cows had the highest milk fat percentage (3.93%), and the Holstein cow in the dairy farm with 1000 to 2000 cows had the lowest milk fat percentage (3.59%). The milk fat percentage of Holstein cows with fifth parity was 4.01%, which was significantly higher than those of the other parities (*p* < 0.01). The milk fat percentage of Holstein cows with first parity was 3.76%, which was significantly lower than those of the other parities (*p* < 0.01). The milk fat percentage of Holstein calving in autumn was 3.88%, which was significantly higher than in other calving seasons (*p* < 0.01). In spring, the milk fat percentage was 3.77%, which was significantly lower than in the other calving seasons (*p* < 0.01). The milk fat percentage of Holstein cattle with a calving interval of more than 441 days was 3.90%, which was significantly higher than those of other calving intervals (*p* < 0.01). When the calving interval was 300 to 365 days, the milk fat percentage was 3.88%, which was significantly lower than the percentage during other calving seasons (*p* < 0.01). The milk fat percentage was significantly higher for Holstein cows with a 305-days milk yield of 3000–5000 kg than in other 305-days milk yield cows (*p* < 0.01).

### 3.2. Effects of Different Factors on Lactation Curve and Fitting Parameters of Daily Milk Fat Percentage

The fitting parameters of the daily milk fat percentage Wood lactation curve with different factors are shown in Table 4. The daily milk fat percentage Wood lactation curve changes were drawn according to the different parameters.

#### 3.2.1. Dairy Farm Size

**Figure 1:**
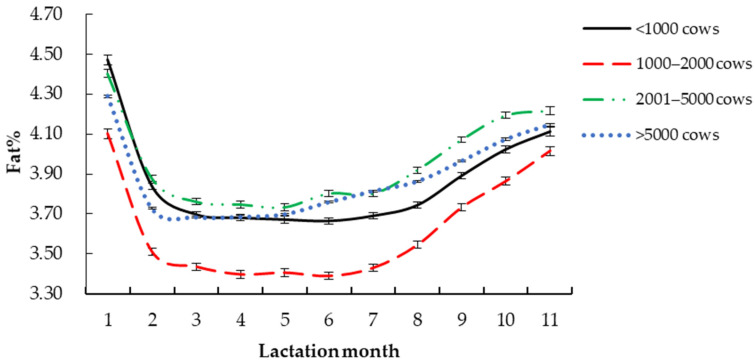
Lactation curves of Wood model for different dairy farm sizes.

The *R*^2^ of the milk fat percentage curve was the highest (0.9798), and the mean square error was the lowest (0.036), on farms with fewer than 1000 cows (Figure 1, Table 4). When the number of dairy cows in the dairy farm was 1000 to 2000, the *R*^2^ of the milk fat percentage curve was the lowest (0.9483). The *Y_min_* of dairy cows was the highest when the number of dairy cows was more than 5000 (3.58%). The *T_min_* of dairy cows with less than 1000 cows in the dairy farm was the highest (142 days). The *Per* of dairy cows with a size less than 1000 was the highest (3.65), while the *Per* of dairy cows with a scale of 1000 to 2000 was the lowest (3.44). The *a* of dairy cows with a size less than 1000 was the highest (4.10). When the number of dairy cows was 1000 to 2000, the *c* and *b* of the lactation curve were the highest, which were 0.07 and 0.3, respectively.

#### 3.2.2. Parity

**Figure 2:**
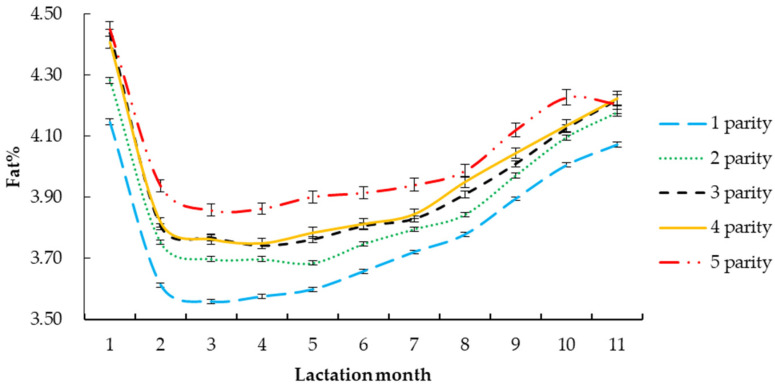
Lactation curves of Wood model for different parities.

The *R*^2^ of the milk fat percentage curve of the first birth of Holstein cattle was the highest (0.9710), the mean square error was the lowest (0.050), and the *R*^2^ of the fifth birth was the lowest (0.9685), as shown in Figure 2 and Table 4. The *Y_min_* of the fifth parity of Holstein cattle was the highest (3.74%). The *T_min_* of Holstein cattle was the highest in the third parity (129 days). The fifth fetus had the highest *Per* of milk fat percentage (3.68). The *a* of the fifth parity of Holstein cattle was the highest (4.07). Among different parities, the *c* of lactation curve of the third and fourth parity was the largest (0.06), and the *b* of the third parity was the largest (0.24).

#### 3.2.3. Calving Season

**Figure 3:**
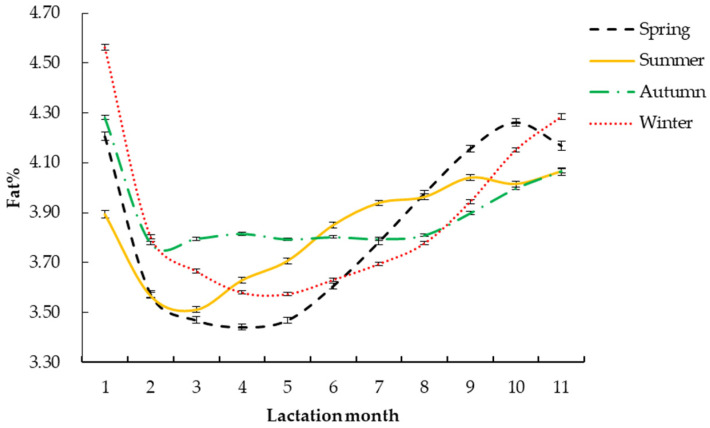
Lactation curves of Wood model for different calving seasons.

The lactation curves and fitting parameters of different calving seasons are shown in Figure 3 and Table 4. Among them, the *R*^2^ of the milk fat percentage curve of calving in autumn was the highest (0.9720). The *Y_min_* of calving in autumn was the highest (3.65%), and that of calving in spring was the lowest (3.36%). The *T_min_* for Holstein calving in autumn was the highest (137 days). The *Per* of calving in spring was the lowest (3.24). The *a* and *b* of the lactation curve of Holstein calving cows in winter were the largest, 4.07 and 0.35, respectively, and the *c* was lowest in autumn (0.03).

#### 3.2.4. Calving Interval

**Figure 4:**
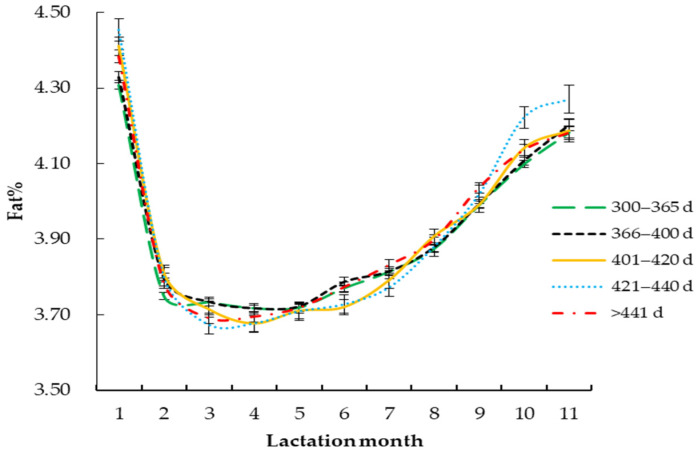
Lactation curves of Wood model for different calving intervals.

The *R*^2^ of the milk fat percentage curve was the highest (0.9704), and the mean square error was the lowest (0.054), when the calving interval of Holstein cattle was 366–400 days (Figure 4 and Table 4). The *Y_min_* was the highest (3.59%) when the calving interval was 366–400 days. The *T_min_* was the highest when the calving interval was 401–420 days (128 days). When the calving interval was 300–365 days, the *Per* was the highest (3.60), and when the calving interval was 421–440 days, the *Per* was the lowest (3.45). The *a* was the highest (4.00) when the calving interval was 421–440 days, and the *a* was the lowest (3.91) when the calving interval was 300–365 days. The maximum speed of *c* and *b* were 0.07 and 0.28, respectively, when the calving interval was 421–440 days.

#### 3.2.5. 305-Day Milk Yield

**Figure 5:**
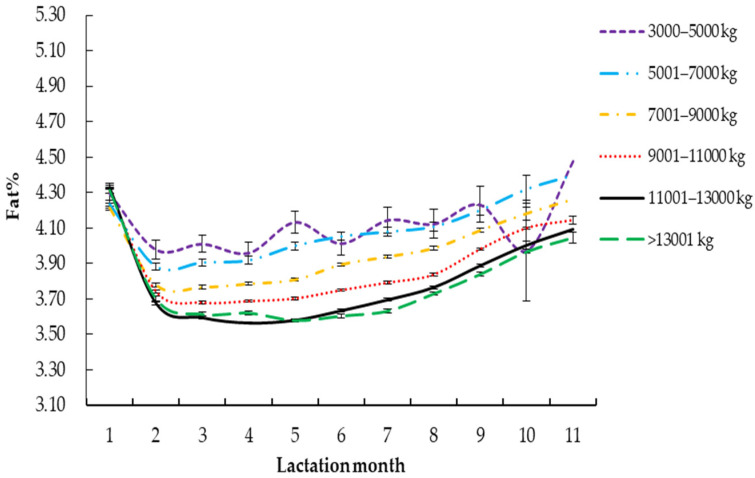
Lactation curves of Wood model for different 305-days milk yield.

As shown in Figure 5 and Table 4, the *R*^2^ of the milk fat percentage curve of Holstein cows with a 305-days milk yield of 5001–7000 kg was the highest (0.9734). The *Y_min_* of Holstein cows with milk yield of 5001–7000 kg was the highest (3.67%). The *T_min_* was the highest when milk yield was 9001–11,000 kg (128 days). Holstein cows with milk yield of 3000–5000 kg had the highest *Per* (4.22), and Holstein cows with milk yield of 11,001–13,000 kg had the lowest *Per* (3.54). The *a*, *c* and *b* of Holstein cattle with milk yield greater than 13,001 kg in 305-days milk yield were the highest, with their corresponding values 4.14, 0.07 and 0.34, respectively.

## 4. Discussion

Wang et al. [15] and Zhang et al. [21] have shown that the size of farm has a significant effect on the parameters of the Holstein lactation curve model. Kuevi et al. [22] found that the size of the dairy farm had a significant effect on the milk fat percentage of dairy cows. This study found that the farm size had a certain effect on the Holstein milk fat percentage Wood lactation curve model parameters. Large-scale dairy farms adopt modern equipment and more meticulous feeding management to shorten the period of milk fat percentage declining before the peak period of cow intake. Therefore, dairy farms with more than 5000 cows have the shortest *T_min_* and the highest milk fat percentage. Related studies have shown that large-scale farm equipment has a high degree of mechanization, and that the establishment of a digital standardized management system can enable various departments to cooperate with each other, to have strong technical strength of operation, and to diversify the types of formulations, which is helpful for improving the lactation performance and efficiency of dairy cows [23,24]. Cows in large-scale dairy farms have been improved by standard and scientific breeding, and their genetic basis and production performance have improved. Holstein cows’ milk fat percentage curve, with a size of less than 1000, has the highest *R*^2^, the greatest *Per*, and the greatest potential of milk fat percentage. Small-scale pastures generally have a low degree of mechanization; although the number of cattle is small, the management of individuals is more thorough. On the other hand, medium-sized farms may be using low mechanization and poor cattle management, because of cost considerations; besides, the DHI records of medium-sized farms are not comprehensive. This may be the reason why 1000–2000 pastures have the lowest *R*^2^, the least *Per*, and the least potential of milk fat percentage. Therefore, the management of medium-sized pastures should be strengthened to ensure lactation performance.

This study found that the *R*^2^ of the fifth parity dairy cow was the smallest, the *Y_min_* was the highest, the *Per* was the highest, and the lactation potential was the greatest. The *R*^2^ of the first parity dairy cow was the highest, the *Y_min_* was the lowest, the *Per* was the lowest, and the potential of the milk fat percentage was the least. Roberto et al. [25] found that the parameters of the population lactation curve fitted by the Wood model were significantly affected by parity, and that the lactation potential of first-born cattle was low. The change of milk fat percentage of the first parity cows was relatively small, and the data were limited, which may be due to the fact that the majority of the first parity cattle had just reached body maturity. The dairy cows’ body development was not perfect, and the nutrient intake required to maintain the dairy cows’ energy consumption and tissue development wass not good, resulting in poor milk fat percentage. For first-born cattle, the potential of milk fat percentage was low, the milk fat percentage increased slowly in the later stage of lactation, and the *Per* was strong, which was consistent with the findings of Rao and Sundaresan [26]. High-parity cows had a small *R*^2^ because some individuals showed superior production performance, and there were significant differences between individuals. Capuco et al. [27] and Val-Arreola et al. [28] found that milk fat percentage increased significantly with the increase of parity, which was consistent with the findings of this study, and that lactation performance reached its peak at the fifth fetal stage. Amongst all the parities, Holstein cattle of the third parity first reached the lowest point of milk fat percentage and then increased the fastest, and this showed that the body recovery ability of dairy cows with third parity was the fastest, such that they could quickly eliminate negative effects after calving, and enter a state of efficient production. Knaus et al. [29] and Oltenacu et al. [30] showed that the average calving number of dairy cows in Austria and the United States was 3.3 when they were eliminated. Yan et al. [31] found that the average calving number of Chinese Holstein cattle was 2.86 when they were eliminated. Therefore, the productive lifetime of the dairy cow should be improved to improve the actual production income.

Qi et al. [32] found that the calving season has a certain influence on the Wood lactation curve model parameters. In this study, different calving seasons had corresponding effects on milk fat percentage and the Wood lactation curve model parameters of Holstein cattle. Among them, the *R*^2^ of calving in autumn, the *Y_min_*, the shortest *T_min_*, and the *Per* were the highest, and the milk fat percentage was the highest; similar results have also been found by Tekerli et al. [33], Keown et al. [34], and Schneeberger [35]. This may be due to the suitable temperature in autumn, when the calving environment is less stressful on dairy cows; coupled with the supplementary feeding of fresh green feed in autumn, the lactation of dairy cattle can be brought into full play. When calving in spring, the *R*^2^, the shortest *T_min_* and the *Per* are the lowest. This could be because the calving cows in spring have just experienced a cold winter and are recovering, when calving increases the burden on the body; on the other hand, there is no high-quality green feed in spring, and the demand for nutrition of dairy cows cannot be met, resulting in low lactation performance. García et al. [36] found that the milk fat content of cows during calving in spring was significantly lower than that in other seasons, which was consistent with the results of this study. The potential of milk fat percentage and the shortest *T_min_* in summer calving cows are the smallest, which may be due to the influence of heat stress during summer calving, which reduces the dry matter intake and adipose tissue mobilization ability of dairy cows [37,38,39,40]; the dairy cow’s need for energy to maintain itself increases, and the energy used to produce milk decreases. However, as dairy cows drink a lot of water due to hot weather, the milk fat percentage decreases rapidly, and the shortest *T_min_* is also the smallest. Therefore, the dairy farm should reasonably arrange the breeding time of the herd to avoid calving in spring as far as possible, to improve the production income.

In this study, it was found that calving intervals had a significant effect on milk fat percentage and the parameters of the Wood lactation curve model: when the calving interval was 366 to 400 days, the *R*^2^ was the highest, and the *Y_min_* was greatest. Related studies [41,42,43] have shown that production performance and production benefit are best when the calving interval of dairy cows is 12–13 months; using the production data at this time to fit, the *R*^2^ is the highest. The lactation potential is minimum when the calving interval is 300–365 days; a short calving interval means that there are more aborted cattle in the herd, and the production performance of the herd decreases [44]. The *R*^2^ of Holstein cattle with a calving interval of more than 441 days was the lowest, and this may have been due to the negative balance of IGF-I [45], the signal factor secreted by the body. The secretion capacity of reproductive hormones decreased while reproductive diseases increased, resulting in longer calving intervals and longer lactation days, so the *R*^2^ of dairy cows was the lowest. Therefore, matching the appropriate calving interval in production will help to improve the economic benefit of breeding.

Baiyila [46] showed that the difference in milk yield over 305 days had a certain effect on the Wood lactation curve model. This study found that the *R*^2^ of Holstein cattle was the highest when the 305-days milk yield was 5001–7000 kg. Among them, the milk fat percentage of Holstein cattle with a 305-days milk yield of 3000–5000 kg was the highest, and it was found that the milk fat percentage decreased significantly with the increase of milk yield. Umphrey et al. [47] and Liang et al. [48] showed a significant negative correlation between milk yield and milk fat percentage. The Holstein cows with 305-days milk yield above 13,001 kg were high-yield dairy cows; the results showed that the lactation potential of this kind of dairy cow was the best, the speed of reaching and rising from the lowest point of milk fat percentage was the highest, and *Y_min_* was the highest. However, related studies [49,50] have shown that high-yield dairy cows may increase their own diseases at the same time as high milk yield, implying that some high-yield dairy cows may get sick, which may affect the milk fat content during lactation, resulting in great individual differences amongst individuals as a whole; this may be the reason for the least *R*^2^. The Holstein cattle with milk yield of 3000–5000 kg reached the lowest point of milk fat percentage at the slowest speed, and the speed of rising from the lowest point was the least, this may be due to the decline of lactation performance in some Holstein cattle due to heredity and disease, and the specific reasons need to be further studied.

## 5. Conclusions

The findings of this study show that the Wood model is suitable for fitting the curve of milk fat percentage of Holstein cattle in Jiangsu Province. Non-genetic factors such as dairy farm scale, calving season, parity, calving interval, and 305-days milk yield have significant effects on milk fat percentage and milk fat percentage curve fitting parameters. This study of non-genetic factors provides a referential basis for regulating and controlling the milk fat percentage of Holstein cattle in Chinese dairy farms.

## Figures and Tables

**Table 1:** Ingredients and nutrient composition of the diet (dry matter basis).

Item	Percentage
Ingredient, % of DM	
Alfalfa hay	25.41
Corn silage	28.40
Oat hay	6.16
Ground corn	17.48
Soybean meal	5.26
Cottonseed meal	4.06
Distillers dried grains with solubles	5.31
Barely	5.17
Limestone	0.32
NaHCO_3_	0.36
NaCl	0.31
CaHPO_4_	0.56
Premix	1.20
Composition, % of DM	
Crude protein	15.02
Ether extract	3.96
Neutral detergent fiber	41.11
Acid detergent fiber	22.04
Calcium	0.82
Phosphorus	0.42
NE_L_, ^1^ Mcal/kg	6.29

The premix provided the following per kg of the concentrate: VA 300000 IU, VD 385000 IU, VE 1455 IU, nicotinic acid 550 mg, Cu 770 mg, Mn 930 mg, Fe 1200 mg, Zn 3600 mg, Se 21 mg, I 50 mg, Co 12 mg. ^1^ NEL was a calculated value according to NRC(2001), while the others were measured values.

**Table 2:** Basic information of milk fat percentage data for dairy cows at each parity.

Parity	Number	Mean	Standard Deviation	Minimum	Maximum
1	157,483	3.76	0.37	1.02	6.93
2	123,340	3.85	0.39	1.11	6.99
3	69,022	3.92	0.40	1.04	6.94
4	28,315	3.94	0.39	1.13	6.83
5	20,289	4.01	0.39	1.03	6.72
Total	398,449	3.90	0.39	1.01	6.98

**Table 3:** Effects of different factors on milk fat percentage (LSM ± SE).

Factor		Number	Milk Fat Percentage
Dairy farm size	<1000	19,189	3.84 ± 0.01 ^B^
	1000~2000	30,020	3.59 ± 0.01 ^C^
	2001~5000	45,853	3.93 ± 0.01 ^A^
	>5000	303,387	3.85 ± 0.00 ^B^
	*F* value		436.331 **
Parity	1	157,483	3.76 ± 0.00 ^D^
	2	123,340	3.85 ± 0.00 ^C^
	3	69,022	3.92 ± 0.00 ^B^
	4	28,315	3.94 ± 0.01 ^B^
	5	20,289	4.01 ± 0.01 ^A^
	*F* value		195.399 **
Calving season	Spring	46,297	3.77 ± 0.00 ^D^
	Summer	53,302	3.81 ± 0.00 ^C^
	Autumn	178,639	3.88 ± 0.00 ^A^
	Winter	120,211	3.83 ± 0.00 ^B^
	*F* value		59.986 **
Calving interval (Days)	300~365	89,259	3.88 ± 0.00 ^B^
	366~400	57,789	3.89 ± 0.00 ^AB^
	401~420	18,525	3.89 ± 0.01 ^AB^
	421~440	12,561	3.89 ± 0.01 ^AB^
	>441	40,154	3.90 ± 0.01 ^A^
	*F* value		129.525 **
305-days milk yield (kg)	3000~5000	2180	4.07 ± 0.02 ^A^
	5001~7000	13,276	4.04 ± 0.01 ^B^
	7001~9000	59,589	3.93 ± 0.00 ^C^
	9001~11,000	125,981	3.84 ± 0.00 ^D^
	11,001~13,000	102,605	3.75 ± 0.00 ^E^
	13,001~15,000	54,960	3.74 ± 0.00 ^E^
	*F* value		382.315 **

In the same factor and column, the superscript does not contain the same capital letters to indicate that the difference is extremely significant (*p* < 0.01); the same letter means no significant difference (*p* > 0.05). ** indicated that the difference reached a highly significant level (*p* < 0.01).

**Table 4:** Wood model fitting parameters of daily milk fat percentage by different factors.

Factor		a	b	c	*T_min_* (month)	*T_min_* (day)	*Y_min_* (%)	*Per*.	*R* ^2^	Residual Mean Squares
Dairy farm size	<1000	4.10 ± 0.02	0.26 ± 0.01	0.06 ± 0.00	4.71	142	3.56	3.65	0.9798	0.036
	1000–2000	3.65 ± 0.02	0.30 ± 0.01	0.07 ± 0.00	4.21	127	3.21	3.44	0.9483	0.084
	2001–5000	3.96 ± 0.01	0.26 ± 0.01	0.06 ± 0.00	4.18	126	3.55	3.51	0.9560	0.082
	>5000	3.89 ± 0.01	0.21 ± 0.00	0.05 ± 0.00	4.08	123	3.58	3.59	0.9737	0.047
Parity	1	3.74 ± 0.01	0.21 ± 0.00	0.05 ± 0.00	3.89	117	3.47	3.54	0.9710	0.050
	2	3.89 ± 0.01	0.22 ± 0.00	0.05 ± 0.00	4.13	124	3.55	3.58	0.9696	0.055
	3	4.02 ± 0.01	0.24 ± 0.00	0.06 ± 0.00	4.29	129	3.61	3.58	0.9701	0.055
	4	3.99 ± 0.01	0.23 ± 0.01	0.06 ± 0.00	4.16	125	3.62	3.56	0.9685	0.059
	5	4.07 ± 0.02	0.19 ± 0.01	0.05 ± 0.00	4.22	127	3.74	3.68	0.9695	0.058
Calving season	Spring	3.68 ± 0.01	0.31 ± 0.01	0.08 ± 0.00	3.65	110	3.36	3.24	0.9674	0.057
	Summer	3.54 ± 0.01	0.11 ± 0.00	0.04 ± 0.00	2.86	86	3.52	3.65	0.9716	0.050
	Autumn	3.95 ± 0.01	0.16 ± 0.00	0.03 ± 0.01	4.56	137	3.65	3.91	0.9720	0.051
	Winter	4.07 ± 0.01	0.35 ± 0.00	0.08 ± 0.00	4.37	132	3.46	3.41	0.9685	0.056
Calving interval (Days)	300–365	3.91 ± 0.01	0.22 ± 0.00	0.05 ± 0.00	4.15	125	3.57	3.60	0.9691	0.056
	366–400	3.94 ± 0.01	0.22 ± 0.00	0.05 ± 0.00	4.13	124	3.59	3.58	0.9704	0.054
	401–420	3.99 ± 0.02	0.26 ± 0.01	0.06 ± 0.00	4.26	128	3.55	3.52	0.9688	0.057
	421–440	4.00 ± 0.02	0.28 ± 0.01	0.07 ± 0.00	4.16	125	3.54	3.45	0.9691	0.057
	>441	3.95 ± 0.01	0.24 ± 0.01	0.06 ± 0.00	4.16	125	3.56	3.53	0.9685	0.058
305-day milk yield (kg)	3000–5000	3.62 ± 0.17	0.00 ± 0.07	0.02 ± 0.01	0.27	9	3.65	4.22	0.9714	0.055
	5001–7000	3.60 ± 0.06	0.05 ± 0.02	0.03 ± 0.00	1.81	55	3.67	3.82	0.9734	0.050
	7001–9000	3.57 ± 0.03	0.09 ± 0.01	0.03 ± 0.00	2.62	79	3.58	3.68	0.9728	0.050
	9001–11,000	3.74 ± 0.03	0.17 ± 0.10	0.05 ± 0.00	3.78	114	3.53	3.63	0.9713	0.051
	11,001–13,000	3.62 ± 0.04	0.18 ± 0.01	0.05 ± 0.00	3.66	110	3.43	3.54	0.9707	0.051
	>13,000	4.14 ± 0.07	0.34 ± 0.02	0.07 ± 0.00	4.79	144	3.43	3.55	0.9699	0.051
Total		3.89 ± 0.00	0.22 ± 0.00	0.05 ± 0.00	4.17	126	3.54	3.59	0.9699	0.054
